# Bioinformatics-Guided Experimental Validation Identifies NQO1 as a Senescence-Ferroptosis Hub in Liver Fibrosis

**DOI:** 10.3390/biomedicines13051249

**Published:** 2025-05-20

**Authors:** Xinying Zhang, Chunmeng Fu, Ziyue Yang, Yue Tan, Huan Li, Xiangqian Zhang, Mengru Chen, Fang Peng, Ning Li

**Affiliations:** 1 Department of Blood Transfusion, Xiangya Hospital, Central South University, Changsha 410008, China; 238111081@csu.edu.cn (X.Z.);; 2 Clinical Transfusion Research Centre, Xiangya Hospital, Central South University, Changsha 410008, China; 3 Department of Clinical Laboratory, Xiangya Hospital, Central South University, Changsha 410008, China; 4 National Clinical Research Center for Geriatric Disorders, Xiangya Hospital, Central South University, Changsha 410008, China; f1734506@163.com; 5 NHC Key Laboratory of Cancer Proteomics, Department of Oncology, Xiangya Hospital, Central South University, Changsha 410008, China; 6 Department of Hepatobiliary Surgery, Liver Transplantation Liver Cancer Institute and Zhongshan Hospital, Fudan University, Shanghai 200032, China; 7 Department of Pharmacology & Pharmaceutical Sciences, University of Southern California, Los Angeles, CA 90089, USA

**Keywords:** liver fibrosis, cellular senescence, ferroptosis, biomarkers, NQO1, immune cells

## Abstract

**Background:** As a pivotal point for the development of liver diseases, liver fibrosis (LF) is closely associated with cellular senescence and ferroptosis. However, there is a lack of effective markers that accurately predict LF status. This study aims to identify key genes involved in LF through bioinformatics analysis and experimental validation. **Methods:** We used bioinformatics analysis of Gene Expression Omnibus (GEO) data to investigate the gene functions, prognostic value, and immune associations of characteristic genes in LF. Functional enrichment analysis of DEGs was performed using GO and KEGG. Immune cell types and their proportions were estimated with CIBERSORTx. In addition, we analyzed the role of NQO1 in LF using IHC, WB, PCR, and flow cytometry. **Results:** Bioinformatics analysis identified 10 hub genes, including *AR, CDKN1A, GJA1, CTSB, HIF1A, HMGB1, NQO1, PARP1, PTEN*, and *TXN*. Among them, *NQO1* was strongly correlated with immune cell activity. Experimental validation confirmed that NQO1 is upregulated and promotes αSMA and COL1A1 expression in hepatic stellate cells (HSCs). Knockdown of *NQO1* significantly affected the proliferation of HSCs. **Conclusions:** NQO1 plays a critical role in HSC senescence and ferroptosis, promoting HSC activation and contributing to LF progression. Our findings suggest that NQO1 may serve as a potential biomarker for LF.

## 1. Introduction

Liver diseases account for 2 million deaths annually, posing a significant global health challenge [1]. Liver fibrosis (LF) is a critical step in the progression of various chronic liver diseases to cirrhosis. It is characterized by dynamic pathological changes with extensive extracellular matrix (ECM) deposition [2]. As the primary cell population responsible for ECM synthesis in the liver, hepatic stellate cells (HSCs) play a pivotal role in the activation, proliferation, and transformation processes during LF. Typically, after liver injury, HSCs become activated and differentiate into myofibroblasts that secrete collagen-rich ECM, leading to excessive ECM accumulation [3]. However, there are currently no clinically approved drugs for the treatment of LF, and its mechanisms are not fully understood. Consequently, targeting activated HSCs has emerged as an alternative antifibrotic strategy.

Cellular senescence is a stable, programmed response characterized by irreversible cell cycle arrest [4]. It can be triggered by various stressors and is marked by features such as permanent cell cycle arrest (upregulation of p53, p16, and p21), senescence-associated secretory phenotypes (SASPs), and increased senescence-associated β-galactosidase activity [5]. Studies have shown that senescent activated HSCs accumulate in cirrhotic livers and limit fibrosis following liver injury [6]. Thus, inducing HSC senescence may act as a novel therapeutic concept for fibrosis treatment [7].

Inducing HSC death has been widely reported to clear activated HSCs and reverse LF [8]. Numerous studies have revealed that LF progression is closely linked to various forms of programmed cell death, such as apoptosis, autophagy, pyroptosis, necroptosis, ferroptosis, cuproptosis, and panoptosis [9,10]. Ferroptosis, a non-apoptotic cell death process, is characterized by intracellular iron accumulation, increased toxic lipid peroxides, and elevated levels of reactive oxygen species. Excessive intracellular iron triggers a cascade of downstream signaling pathways with self-destructive effects [11]. Studies have shown that fibroblast growth factor 21 alleviates iron overload-induced liver injury and fibrosis by inhibiting ferroptosis [12]. Sorafenib reduced fibrosis by inducing HSC ferroptosis via the hypoxia-inducible factor (HIF)-1α/SLC7A11 signaling pathway in a mouse LF model [13].

In summary, we aim to explore alternative strategies for alleviating LF by focusing on HSC senescence and ferroptosis. Through bioinformatics analysis, animal experiments, and clinical sample validation, we will investigate the key regulatory genes linking these processes to identify the potential mechanisms underlying fibrosis progression. This study aspires to discover improved biomarkers for the treatment of LF.

## 2. Materials and Methods

### 2.1. Data Collection and Gene Identification

In this study, gene expression microarray data for LF samples were primarily obtained from the GEO database (https://www.ncbi.nlm.nih.gov/geo/, accessed on 12 May 2025). We downloaded the dataset GSE25097, which includes 6 normal human liver tissue samples and 40 LF samples. To identify differentially expressed genes (DEGs), we compared the LF group with the normal group in the GSE25097 dataset using the limma R package in R version 4.4.0, selecting genes with a |log2 fold change (FC)| > 1 and *p* < 0.05. Using “senescence” as the keyword, aging-related genes were downloaded from the GenAge (The Ageing Gene Database) (https://genomics.senescence.info/genes/index.html, accessed on 12 May 2025). Ferroptosis-related gene data were obtained from the FerrDb database (http://www.zhounan.org/ferrdb, accessed on 12 May 2025). Finally, by intersecting the DEGs with the aging-related and ferroptosis-related gene sets, we identified a subset of genes associated with all three categories for further analysis.

### 2.2. Gene Enrichment Analysis of Differential Genes in LF-Aging-Ferroptosis

We performed functional enrichment analysis on DEGs from three datasets. Gene Ontology (GO) and Kyoto Encyclopedia of Genes and Genomes (KEGG) pathway analyses were conducted to explore the potential roles of these genes in biological processes, cellular components, molecular functions, and key signaling pathways [14]. GO and KEGG enrichment analyses were performed using the “clusterProfiler” package in R (version 4.4.0), with a significance threshold set at *p* < 0.05. Finally, the top 10 enriched GO and KEGG terms were visualized using the SRplot web server (http://www.bioinformatics.com.cn/SRplot, accessed on 12 May 2025), which provides interactive and customizable plotting tools for biological data [15].

### 2.3. Identification of hub DEGs

To further investigate the interactions between proteins, we utilized the STRING tool (https://cn.string-db.org/) to construct a protein–protein interaction (PPI) network of the DEGs. To improve the reliability of screening hub DEGs, we applied five commonly used algorithms to evaluate and select hub DEGs: degree, closeness, betweenness, maximum neighborhood component, and bottleneck [16,17]. Subsequently, we employed the GeneMANIA platform (http://genemania.org/) to build a co-expression network of these key DEGs, providing additional support for further research [18].

### 2.4. Immune Infiltration Analysis

We used the CIBERSORTx tool (https://cibersortx.stanford.edu/, accessed on 12 May 2025, version 1.03) to analyze the proportions of 22 immune cell types in tissue samples [19]. To better illustrate the correlations between different immune cells, we visualized the results using a heatmap. Additionally, we used a forest plot to intuitively present the correlations between each hub DEG and immune cell abundance.

### 2.5. Molecular Docking

To investigate potential drug–protein interactions, we conducted molecular docking analysis using the CB-Dock2 platform (https://cadd.labshare.cn/cb-dock2/index.php, accessed on 12 May 2025). The dimensional structure of the target protein was retrieved from the RCSB PDB database (https://www.rcsb.org/), while candidate small-molecule compounds were obtained from the PubChem database (https://pubchem.ncbi.nlm.nih.gov/). These structural files were uploaded to the CB-Dock2 web server for automated docking.

### 2.6. Animal Models

Six-week-old male C57BL/6 mice were obtained from the Animal Institute of Central South University. These mice were then randomly divided into three experimental groups: an olive oil control group, a CCL4-treated group (6 weeks), and a CCL4-treated group (8 weeks), with 5–8 mice per group. Prior to each gavage, the mice were weighed and administered 5 μL of a 25% CCL4 solution per gram of body weight. The control group received an equivalent volume of olive oil. Gavage was performed three times a week for the designated treatment periods. Following the treatment, the mice were euthanized, and liver tissues were harvested for further analysis. All animal procedures were approved by the Laboratory Animal Ethics Committee of Xiangya Hospital and complied with ARRIVE guidelines.

### 2.7. Cell Culture

The human HSC LX-2 cell line (CL-0560) was sourced from Procell Life Science (Wuhan, China). Cells were cultured in Dulbecco’s Modified Eagle Medium (DMEM) (Gibco, MA, USA, 11965092), supplemented with 10% fetal bovine serum (FBS, NEWZERUM, Christchurch, New Zealand) and 1% antibiotic solution (Coolaber, Beijing, China). Cultures were incubated at 37 °C in a 5% CO_2_ atmosphere. The cell line was authenticated via short tandem repeat (STR) profiling, and testing confirmed the absence of mycoplasma contamination.

### 2.8. Antibodies and Reagents

Etoposide was obtained from Selleck Chemicals (Houston, TX, USA). Antibodies used were as follows: NQO1 (ET1702-50, ZENBIO, Chengdu, China), α-SMA (19245S, Cell Signaling Technology, Danvers, MA, USA), COL1A1 (72026S, Cell Signaling Technology), p53 (9282T, Cell Signaling Technology), p21 (2947T, Cell Signaling Technology), and β-Tubulin (YM3037, Immunoway, Suzhou, China).

### 2.9. RNA Interference and Transfection

Human NQO1 siRNA was purchased from HEMA (Dongguan, China). siRNA transfections were carried out using jetPRIME (Polyplus, Strasbourg, France, 101000027) according to the manufacturer’s protocol, with 50 μM of siRNA added per well in a 6-well plate. The siRNA sequences are provided in Supplementary Table S6.

### 2.10. Real-Time Fluorescence Quantitative PCR (RT-qPCR)

Total RNA was isolated from cells or liver tissues using Trizol reagent (TAKARA, Shiga, Japan), followed by cDNA synthesis with HiScript III RT SuperMix (Vazyme, Nanjing, China). Quantitative real-time PCR was performed with ChamQ Universal SYBR qPCR Master Mix (Vazyme). Gene expression levels were calculated using the 2^−ΔΔCT^ method, and the mRNA levels were normalized to the control group, which was assigned a value of 1. The primer sequences are listed in Supplementary Table S2.

### 2.11. Western Blot (WB)

Proteins from liver tissues and cells were washed three times with cold 1× PBS and extracted using 1× RIPA buffer (Thermo, Waltham, MA, USA) supplemented with a protease inhibitor cocktail. Protein concentrations were determined using the BCA Protein Assay Kit (Yeasen Biotechnology (Shanghai) Co., Ltd., Shanghai, China). Equal amounts of protein (20–40 µg per sample) were subjected to SDS-PAGE (Bio-Rad, Hercules, CA, USA) and transferred onto polyvinylidene fluoride (PVDF) membranes (Bio-Rad). After blocking with 5% non-fat milk for 1 h, the membranes were incubated overnight at 4 °C with primary antibodies. The membranes were then incubated with horseradish peroxidase-conjugated secondary antibodies for 1 h at room temperature. Protein bands were visualized using chemiluminescence on a Syngene G:BOX (Iselin, NJ, USA), and band intensity was analyzed with ImageJ software (version 1.8.0.345).

### 2.12. Histological Analysis

Human and animal liver tissues were fixed in formalin for 24 h and subsequently embedded in paraffin. Paraffin-embedded tissues were sectioned into 5 µm thick slices for histopathological evaluation. The sections were stained with hematoxylin and eosin (H&E) as well as Picro Sirius Red (PSR) to assess tissue morphology and fibrosis. For immunohistochemistry (IHC), the sections were incubated overnight at 4 °C with primary antibodies and visualized using 3,3′-diaminobenzidine (DAB) as the chromogenic substrate. Staining intensity and distribution were evaluated by two independent pathologists. All human sample experiments were approved by the Clinical Medical Ethics Committee of Xiangya Hospital, Central South University.

### 2.13. Immunofluorescence Staining

Cells cultured on confocal dishes (Nest, Catalog No. N801002) were fixed with 4% paraformaldehyde for 30 min, permeabilized with 0.2% Triton X-100 for 5 min, and blocked with 5% BSA for 1 h. After fixation, the cells were incubated overnight with primary antibodies at 4 °C, followed by incubation with fluorescently labeled secondary antibodies for 1.5 h at room temperature. Nuclei were stained with DAPI, and images were captured using a Zeiss confocal microscope (Zeiss, Jena, Germany).

### 2.14. Cell Cycle Analysis

After cells were harvested using trypsin, they were washed twice with PBS and fixed in 70% ethanol overnight at 4 °C. The fixed cells were then treated with RNase A and subsequently stained with propidium iodide for 30 min at room temperature in the dark. Flow cytometry was used to analyze the stained cells, and PI fluorescence was measured to determine DNA content. Cells in the G1 phase exhibited 2N DNA content, those in the S phase showed intermediate DNA content, and cells in the G2/M phase exhibited 4N DNA content. The percentage of cells in each cell cycle phase was calculated using ModFit software (version 5.0).

### 2.15. Senescence Associated β-Gal Staining (SA-β-Gal Staining)

For imaging analysis, the culture medium was removed from the processed cells, followed by two PBS washes. Cells were fixed with a fixative solution for 15 min and washed twice more with PBS for 5 min each. The staining solution was then added, and the cells were incubated at 37 °C in a non-CO_2_ incubator, protected from light, for 2 to 24 h. After incubation, the staining solution was removed and replaced with PBS. Images were captured using an inverted microscope.

### 2.16. Statistical Analysis

Statistical analyses were conducted using GraphPad Prism 8.0. Data from three independent experiments are presented as mean ± SEM. Normality and homogeneity of variance were verified. For comparisons between two groups, an unpaired Student’s *t*-test was used, while a one-way ANOVA with the Student–Newman–Keuls post hoc test was applied for multiple group comparisons. Kaplan–Meier survival curves were assessed using the log-rank test. Statistical significance was considered at *p* < 0.05, and “ns” denotes no significant difference.

## 3. Results

### 3.1. Enrichment Analysis of Genes Related to LF, Aging, and Ferroptosis

**Figure 1:**
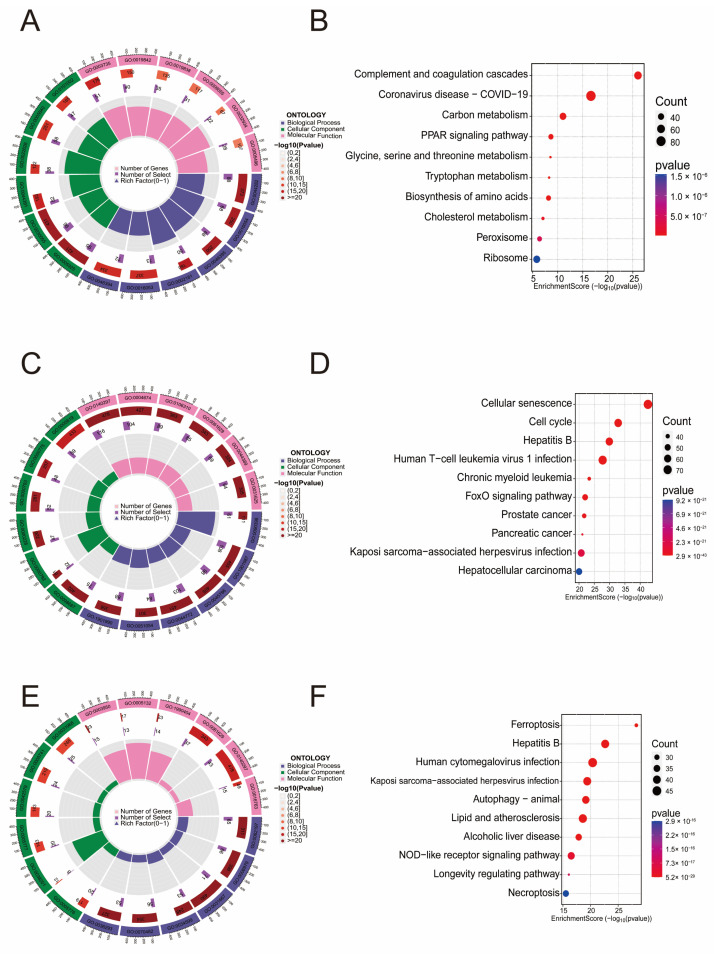
Enrichment analysis of genes related to liver fibrosis (LF), aging, and ferroptosis. (**A**,**B**) GO and KEGG enrichment analysis of LF-related genes. (**C**,**D**) GO and KEGG enrichment analysis of aging-related genes. (**E**,**F**) GO and KEGG enrichment analysis of ferroptosis-related genes.

To investigate the molecular mechanisms underlying liver fibrosis (LF), we analyzed transcriptomic data from the GEO dataset (GSE25097) and identified 1849 differentially expressed genes (DEGs) (Supplementary Table S1). Functional enrichment analysis of these DEGs revealed their involvement in 1424 biological processes (BP), 150 cellular components (CC), and 184 molecular functions (MF) (Supplementary Table S2). Notably, the most significantly enriched terms were associated with immune response and stress response (Figure 1A,B). KEGG pathway analysis further highlighted 94 significantly dysregulated pathways (*p* < 0.05), including the AMPK signaling pathway and ferroptosis.

Additionally, based on the GenAge database, we conducted GO and KEGG enrichment analysis of 866 aging-related genes (correlation score > 1) (Figure 1C,D). The significant results from the GO analysis included 2989 BP terms, 147 CC terms, and 206 MF terms. KEGG enrichment analysis identified 147 significant pathways (*p* < 0.05), including the p53 signaling pathway, IL-17 signaling pathway, and TGF-β signaling pathway (Supplementary Table S3). Enrichment analysis of 454 ferroptosis-related genes also revealed strong correlations (Figure 1E,F) (Supplementary Table S4).

### 3.2. Identification of Hub DEGs Related to LF, Aging, and Ferroptosis

**Figure 2:**
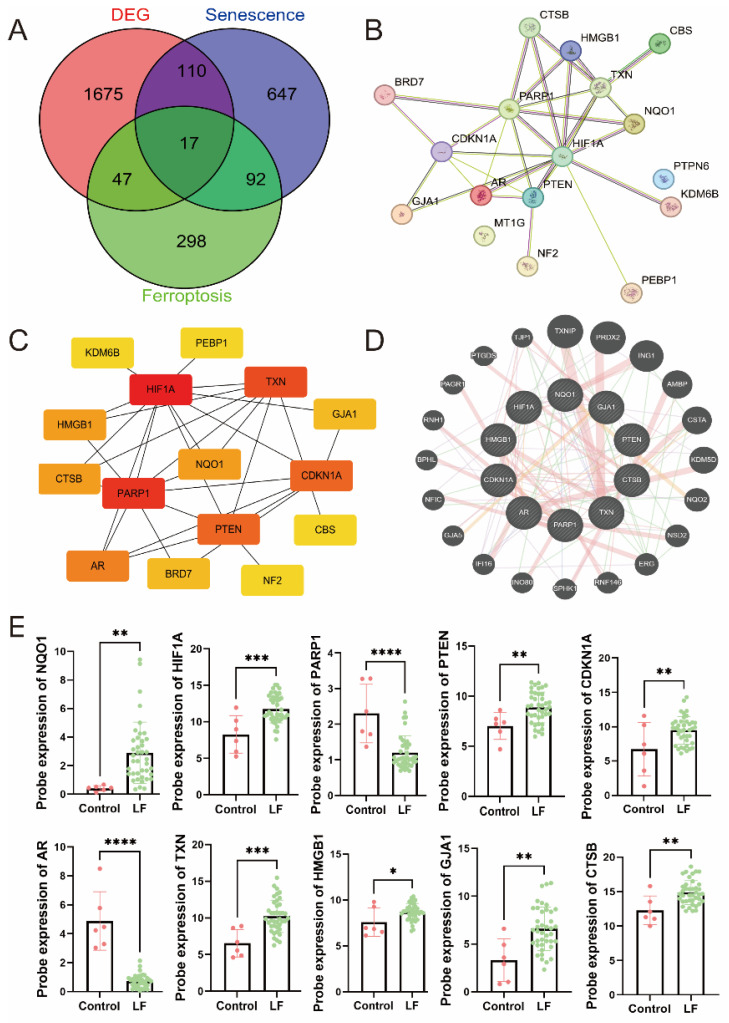
Identification and analysis of hub DEGs. (**A**) A Venn diagram of DEGs related to liver fibrosis, aging, and ferroptosis. (**B**,**C**) The protein–protein interaction (PPI) network of the DEGs. (**D**) Co-expression network of hub DEGs. Co-expression network of hub DEGs: different line types represent networks, while varying pie chart colors indicate distinct functions. (**E**) Probe the expression value of AgDEGs in the LF group and the control group of GSE25097. * *p* < 0.05, ** *p* < 0.01, *** *p* < 0.001, **** *p* < 0.0001.

We identified 17 common DEGs, including *AR, BRD7, CBS, CDKN1A, CTSB, GJA1, HIF1A, HMGB1, KDM6B, NF2, NQO1, PARP1, PEBP1, PTEN, PTPN6, RB1,* and *TXN* (Figure 2A) (Supplementary Table S5). A protein–protein interaction (PPI) network was constructed for these genes (Figure 2B). Using the cytoHubba plugin, we systematically identified the top 10 hub genes through five distinct topological algorithms (Figure 2C). Further network analysis using GeneMANIA revealed a tightly interconnected co-expression network among the most prominent hub genes: *AR, CDKN1A, GJA1, CTSB, HIF1A, HMGB1, NQO1, PARP1, PTEN,* and *TXN* (Figure 2D). The analysis results indicated that these genes are primarily involved in the cell cycle and oxidative stress response.

Next, we further analyzed the expression patterns of hub DEGs. The results showed that, compared to normal tissues, most of the hub DEGs were significantly upregulated in LF tissues (Figure 2E). This suggests that these genes play a key role in the occurrence and progression of LF.

### 3.3. The Relationship Between Hub DEGs and Immune Cells

**Figure 3:**
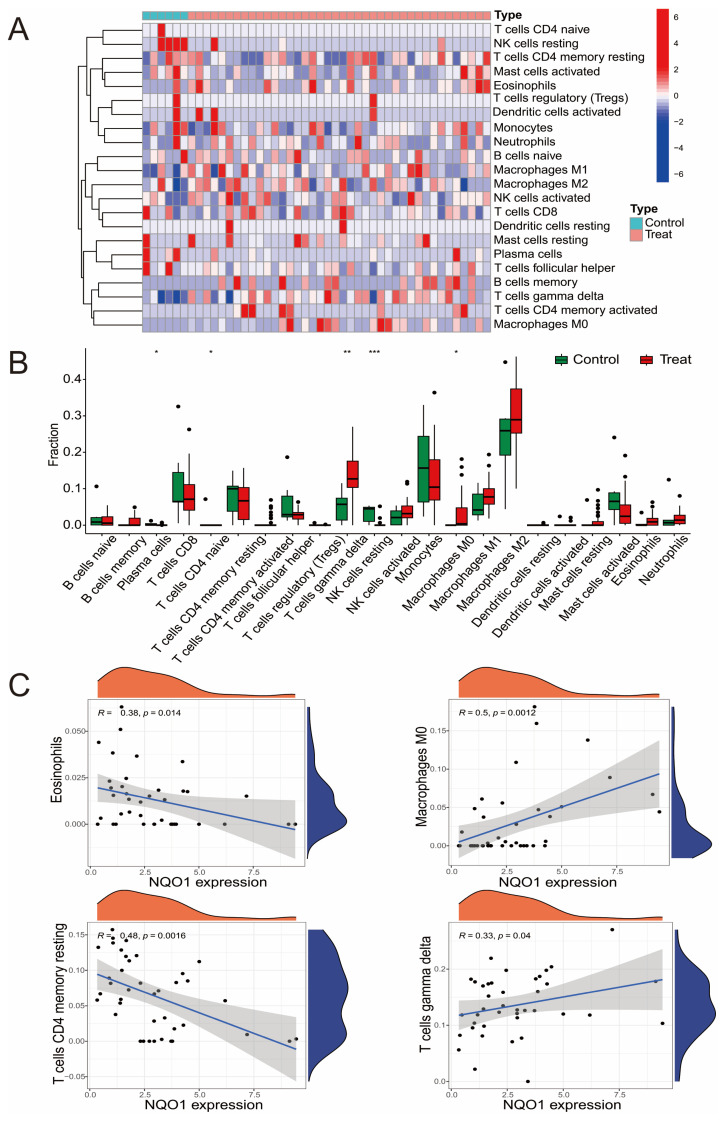
The correlation between the hub DEGs and immune cells. (**A**) The heatmap shows a correlation matrix of 22 immune cell types in LF tissues. The heatmap shows pairwise correlation coefficients between immune cell types, with red representing positive correlations and blue indicating negative correlations. (**B**) The proportion of immune cells in the LF and control groups. * *p* < 0.05, ** *p* < 0.01, *** *p* < 0.001. (**C**) The correlation between hub DEG NQO1 and four types of immune cells.

The previous enrichment analysis highlighted a strong association between DEGs and immune responses. To further explore the immune infiltration characteristics in LF tissue, we employed the CIBERSORTx algorithm to examine the immune cell profiles in LF and control samples. A heatmap was generated to illustrate the correlations among 22 immune cell types (Figure 3A). Additionally, a scatter plot was used to display the differences in immune cell abundance between the LF and control groups (Figure 3B). The analysis revealed that there was a significant increase in M2 macrophages in LF samples, as well as an upward trend in monocytes, CD4 memory resting cells, CD8 T cells, and M1 macrophages. Conversely, the proportions of dendritic cells, B cells, and regulatory T cells (Tregs) were notably reduced (Figure 3C) (Supplementary Figure S1). These findings suggest that the progression of LF is closely associated with disruptions in immune function.

Next, we investigated the relationship between the expression of hub DEGs and the abundance of immune cells. The results indicated that *HMGB1, NAD(P)H:quinone oxidoreductase 1 (NQO1), PTEN,* and *TXN* were positively correlated with gamma delta T cells. *HMGB1* and *NQO1* showed a positive correlation with the abundance of M0 macrophages, while *HMGB1* was positively correlated with activated NK cells. Additionally, *HMGB1, NQO1*, and *HIF1A* were negatively correlated with the proportion of memory resting cells, while *CDKN1A, HMGB1,* and *PTEN* showed negative correlations with regulatory T cells (Tregs) (Supplementary Figure S1).

### 3.4. Clinical Relevance of NQO1

**Figure 4:**
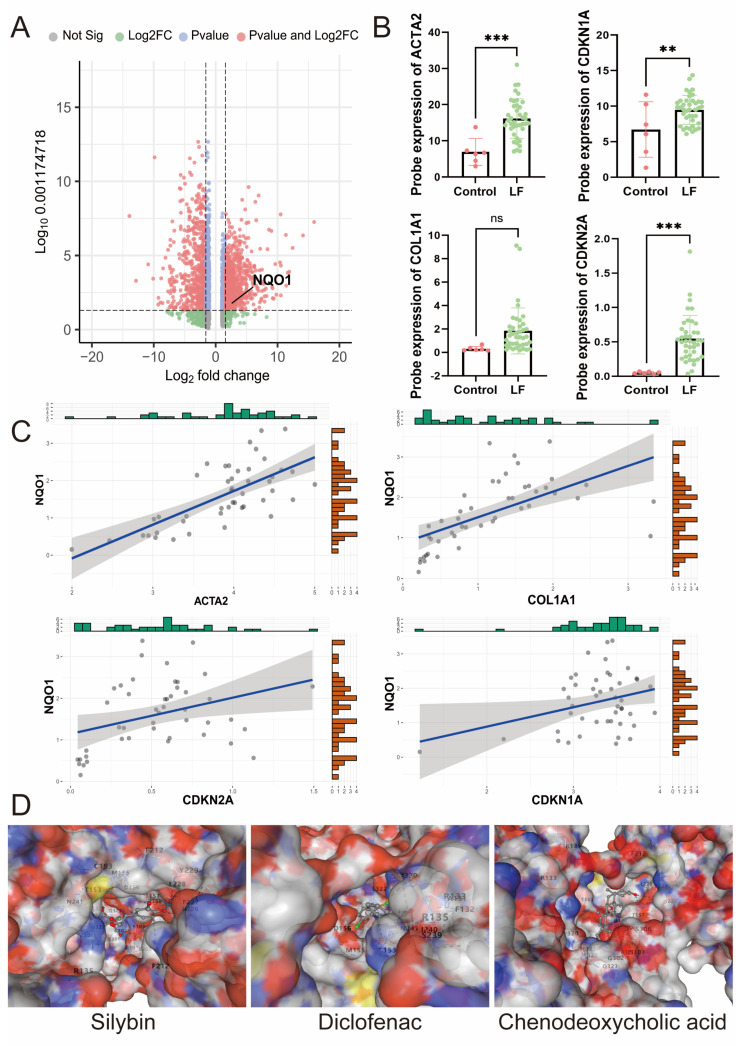
NQO1 expression is increased in liver fibrosis and facilitates the expression of fibrosis markers. (**A**) Volcano plot illustrating DEGs between the LF group and the control group of GSE25097. Red dots represent significantly upregulated genes, while green dots indicate significantly downregulated genes. NQO1 is prominently upregulated in the LF group. (**B**) Probe expression value of ACTA2, COL1A1, CDKN1A, and CDKN2A in the LF group and control group of GSE25097. ns: *p >* 0.05, ** *p* < 0.01, *** *p* < 0.001. (**C**) Correlation analysis of ACTA2, COL1A1, CDKN1A, and CDKN2A with NQO1 in the LF group and control group of GSE25097. (**D**) Molecular docking between NQO1 and different drugs, silybin, diclofenac, and chenodeoxycholic acid.

Given the significant upregulation of *NQO1* during the progression of LF and its critical role in immune infiltration, we conducted a more in-depth clinical analysis of *NQO1* (Figure 4A). [20] According to existing literature, α-SMA and COL1A1 are considered classical markers of HSC activation in LF, while CDKN1A and CDKN2A are markers of HSC senescence. In our study, we found that these markers were significantly upregulated during LF (Figure 4B,C). Further analysis revealed a significant correlation between NQO1 expression and the levels of these markers, suggesting that NQO1 may be closely involved in the progression of LF by regulating both HSC activation and senescence. These findings provide new insights into the potential role of NQO1 as a biomarker in LF.

To investigate the potential of NQO1 as a target for therapeutic strategies, we delved into Genecards to identify the molecular binding sites between NQO1 and three specific drugs. A binding energy value lower than −5 kcal/mol is indicative of a more robust binding activity [21]. The molecular docking results showed that the binding energies of silybin, diclofenac, and chenodeoxycholic acid with STAT3 were −9.5 kcal/mol, −6.6 kcal/mol, and −7.5 kcal/mol. (Figure 4D) The results suggested that NQO1 has potential as a therapeutic target.

### 3.5. The Expression of NQO1 Is Elevated in LF

**Figure 5:**
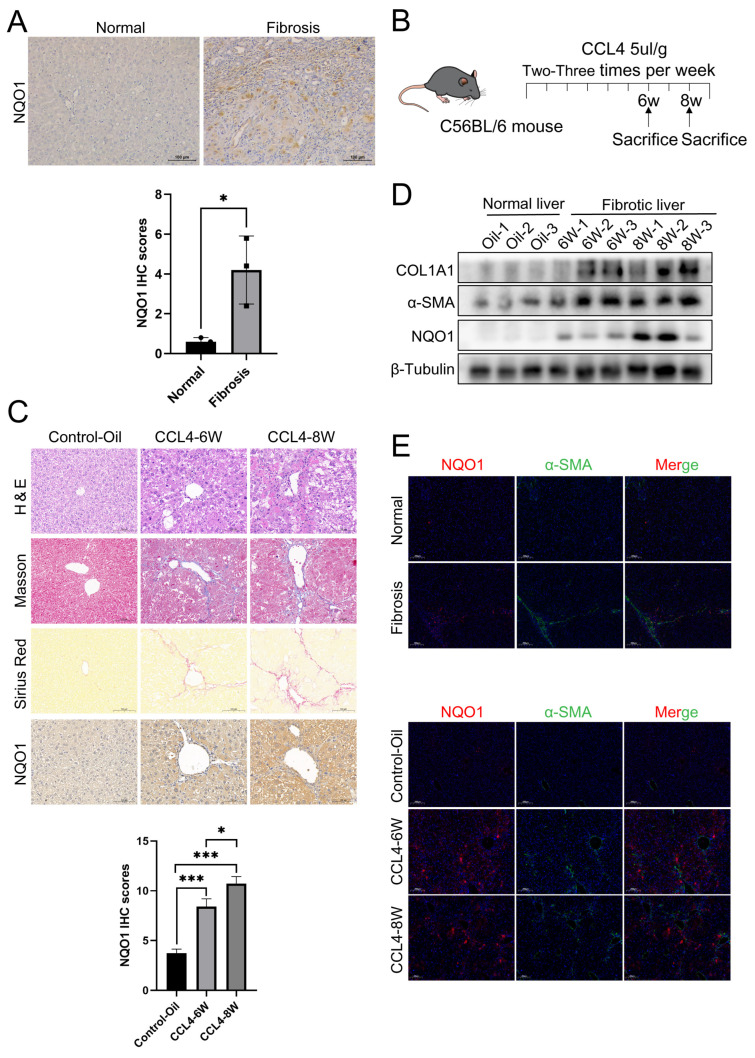
The expression of NQO1 is elevated in LF. (**A**) Representative IHC images showing NQO1 expression in patients with hepatic hemangioma and LF. Scale bar, 100 μm. (**B**) Flowchart of the LF model using CCL4 (carbon tetrachloride). (**C**) IHC images for NQO1, H&E, Sirius red, and Masson staining, along with liver tissue anatomy from control-Oil, 6w-CCl4, and 8w-CCl4 mice (n = 5/group). Scale bar, 100 μm. (**D**) Western blot for NQO1, α-SMA, and COL1A1 in liver tissues from control-Oil, 6w-CCl4, and 8w-CCl4 mice. (**E**) Immunofluorescence staining showing elevated NQO1 (red) and α-SMA (green) in liver tissue from patients and mice (control-Oil, 6w-CCl4, and 8w-CCl4). Scale bar, 200 μm. Data are expressed as mean ± SD. Statistical analysis was performed using Student’s *t*-test. * *p* < 0.05, *** *p* < 0.001.

To further explore the role of NQO1 in LF, we first analyzed its expression in the liver tissues of clinical LF patients. We found that NQO1 expression was elevated in fibrotic tissues (Figure 5A). Next, we validated the expression of NQO1 at the animal level. Based on literature reports, we established a chronic LF model induced by CCl_4_, and collected mouse liver samples at the experimental endpoint [22]. In animal models, six weeks represent mild liver fibrosis, while eight weeks indicate severe liver cirrhosis; they highlight the transition from initial liver injury and fibrosis to advanced cirrhosis. To closely monitor these stages, we performed pathological staining, including Masson’s trichrome, Sirius red, and H&E staining, with results shown in the figures. Strong positive staining was observed in both the 6-week and 8-week groups for Masson’s trichrome, Sirius red, and H&E, indicating significant fibrosis (Figure 5C). Furthermore, we analyzed NQO1 expression in mouse liver tissues using immunohistochemistry (Figure 5C). The results showed strong brown staining in the 6-week and 8-week groups, indicating upregulation of NQO1 expression in the fibrotic livers of mice.

Additionally, we performed WB to assess NQO1 expression in the LF mouse model. The results showed that the expression of fibrosis markers α-SMA and COL1A1 increased progressively with disease progression, and NQO1 exhibited a similar upregulation, with significantly higher expression in both the 6-week and 8-week groups (Figure 5D).

To further investigate NQO1 expression and localization, we conducted immunofluorescence staining for α-SMA and NQO1 in both LF patients and mouse models. Our results revealed a significant increase in the expression of both α-SMA and NQO1 in these fibrosis models. The elevated expression of NQO1, in association with α-SMA, suggests a potential role for NQO1 in the activation and transformation of HSCs into myofibroblasts during LF (Figure 5E).

In summary, these results indicate that NQO1 is highly expressed in both LF patients and the mouse model, and its expression increases progressively with the progression of the disease. This finding suggests that NQO1 could serve as an important modulator of LF and may become a key target for future treatment of LF.

### 3.6. NQO1 Knockdown Induces Senescence and Suppresses LF

**Figure 6:**
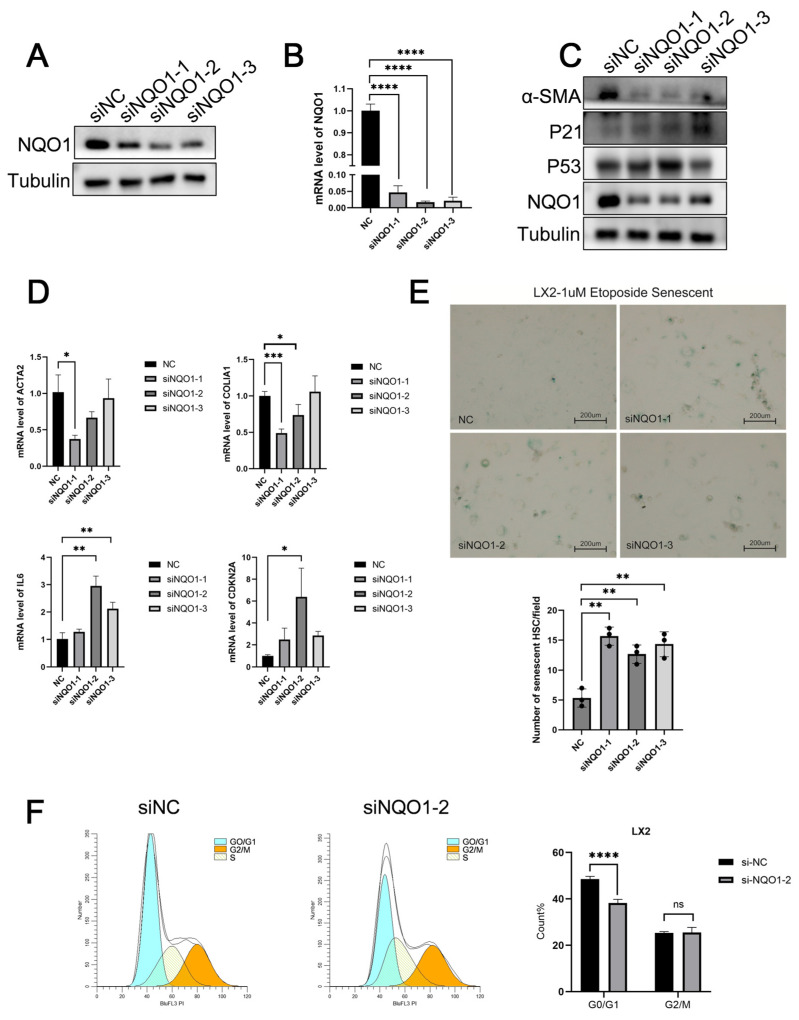
*NQO1* knockdown induces senescence and suppresses LF. (**A**) Western blot analysis showing the efficiency of NQO1 siRNA knockdown in LX-2 cells. (**B**) qPCR analysis confirming the knockdown efficiency of NQO1 siRNA in LX-2 cells. (**C**) Western blot analysis assessing the expression levels of NQO1, α-SMA, COL1A1, p53, and p21 in LX-2 cells after *NQO1* knockdown. (**D**) qPCR analysis of gene expression levels of ACTA2, α-SMA, COL1A1, IL-6, and CDKN2A in LX-2 cells with *NQO1* knockdown. (**E**) Representative images of SA-β-GAL staining in LX-2 cells transfected with negative control (NC) and si-NQO1, along with corresponding statistical analysis of senescence-associated β-galactosidase activity. (**F**) Flow cytometry-based cell cycle analysis of LX-2 cells transfected with NC or si-NQO1, with statistical analysis of cell cycle distribution. All data are presented as mean ± SD. Statistical significance was determined using Student’s *t*-test. * *p* < 0.05, ** *p* < 0.01, *** *p* < 0.001, **** *p* < 0.0001.

To further investigate the role of NQO1 in LF, we conducted in vitro experiments using LX2 cells, where NQO1 expression was knocked down, and the knockdown efficiency was confirmed through WB (Figure 6A) and qPCR (Figure 6B). Among the different siRNA constructs, the second siRNA (siNQO1-2) showed the most effective knockdown effect. Following *NQO1* depletion, we observed a significant downregulation of key LF markers, including α-SMA and COL1A1 proteins (Figure 6C).

Previous studies have demonstrated that cellular senescence can inhibit the activation of hepatic stellate cells, which play a pivotal role in LF [6]. To explore whether *NQO1* knockdown affects cellular senescence, we assessed the expression levels of senescence markers, including p53 and p21. Our results indicated that *NQO1* knockdown led to a significant upregulation of p53 and p21 (Figure 6C), suggesting that NQO1 regulates cellular senescence, potentially contributing to the alleviation of LF. Further qPCR analysis revealed an increase in the expression of SASPs (Figure 6D), aligning with the WB results. Additionally, we performed SA-β-gal staining and cell cycle analysis. The results showed that SA-β-gal staining was significantly enhanced following *NQO1* knockdown (Figure 6E), while cell cycle analysis indicated a G1-phase arrest (Figure 6F). Taken together, these findings suggest that NQO1 may inhibit the occurrence and development of LF by modulating cellular senescence.

## 4. Discussion

Existing research has indicated a rise in immune cell infiltration during the progression of fibrosis [23,24]. To explore the impact of these cells on the immune microenvironment, we discovered a strong correlation between these hub DEGs and macrophages, natural killer (NK) cells, regulatory T cells (Tregs), and memory cells. These results strongly suggest a profound crosstalk between fibrosis and immune modulation. Immune cells, through chronic inflammation, might be the driving force behind the continuous development of fibrosis.

Our in-depth analysis of NQO1 revealed its high expression in LF and its role in inhibiting cellular senescence. NQO1 is a two-electron reductase responsible for quinone detoxification and activation, currently recognized as a therapeutic target for cancer [25]. Studies suggest that NQO1 upregulation in HSCs may promote senescence by reducing reactive oxygen species (ROS) production, alleviating oxidative damage, which prevents malignant transformation and excessive proliferation [26,27]. Considering the central role of HSCs in the development of fibrosis, this finding brings to light a crucial question: the balance between senescence and regeneration within the fibrotic liver [28]. On one hand, cellular senescence can act as a protective mechanism, halting further cell proliferation and the progression of fibrosis [29]. On the other hand, it may also lead to a decline in the tissue’s regenerative capacity [30]. Therefore, NQO1 is likely to play a dual-function role. It not only safeguards the cellular integrity of HSCs but also exerts a regulatory effect on the overall regenerative processes of the liver.

NQO1 also plays a pivotal role in ferroptosis [31]. By catalyzing quinone reduction, NQO1 reduces ROS production and inhibits ferroptosis [32]. In HSCs, iron overload generates excessive ROS via the Fenton reaction, inducing ferroptosis and exacerbating liver injury [33]. Although the upregulation of NQO1 can counteract the damage caused by ROS and inhibit ferroptosis, we must also take into account the complex interactions between ferroptosis and other forms of cell death in the liver, such as apoptosis and necrosis [34]. For instance, merely inhibiting ferroptosis may not be sufficient to completely reverse the fibrotic process. This is especially true when other pathways, like apoptosis, remain active and continue to promote liver injury [35,36]. These complex mechanisms imply that a therapeutic strategy solely centered around NQO1 may need to be integrated into a more comprehensive approach. This comprehensive strategy should target various forms of cell death to effectively impede the development of LF.

Despite the strengths of our integrative analysis, certain limitations should be acknowledged. The “normal” controls in the GSE25097 dataset may represent non-cirrhotic liver tissue rather than truly healthy donor samples; these tissues might already exhibit subclinical pathological alterations. Therefore, this limitation should be taken into account when interpreting the differential gene expression analyses.

In summary, NQO1 may mitigate LF by promoting HSC senescence and inhibiting ferroptosis. When NQO1 levels are reduced, HSCs become more prone to oxidative stress and iron overload, thereby exacerbating fibrosis progression. Thus, NQO1 is regarded as a critical biomarker in LF, presenting a promising target for future therapeutic strategies. Given the complexity of LF mechanisms, further research is needed to fully elucidate the role of NQO1 and develop more effective treatment modalities.

## Data Availability

The data supporting the findings of this study are available from the corresponding author upon reasonable request. Public datasets were retrieved from the GEO database (e.g., GSE25097), the Ageing Gene Database (GenAge), and the FerrDb database.

## Supplementary Materials

The following supporting information can be downloaded at: https://www.mdpi.com/article/10.3390/biomedicines13051249/s1, Figure S1: The correlation between *NQO1* and immune cells. (* *p* < 0.05, ** *p* < 0.01); Figure S2: The correlation between *AR* and immune cells; Figure S3: The correlation between *CDKN1A* and immune cells; Figure S4: The correlation between *GJA1* and immune cells; Figure S5: The correlation between *CTSB* and immune cells; Figure S6: The correlation between *HIF1A* and immune cells; Figure S7: The correlation between *HMGB1* and immune cells; Figure S8: The correlation between *PARP1* and immune cells. Figure S9: The correlation between *PTEN* and immune cells; Figure S10: The correlation between *TXN* and immune cells. Table S1: List of differentially expressed genes (DEGs) identified from the GSE25097 dataset; Table S2: Functional enrichment analysis of DEGs identified from the GSE25097 dataset; Table S3: Functional enrichment analysis of aging-related genes from the GenAge database; Table S4: Functional enrichment analysis of ferroptosis-related genes from the FerrDb database; Table S5: Lists of DEGs from three datasets and their 17 shared genes; Table S6: The list of siRNA sequence and primers sequence.

## Abbreviations

The following abbreviations are used in this manuscript.

BP	biological process
CC	cellular component
CCl_4_	carbon tetrachloride
DAB	diaminobenzidine
DEGs	differential Expressed Genes
ECM	extensive extracellular matrix
GEO	Gene Expression Omnibus
GWAS	genome-wide association study
GO	Gene Ontology
H&E	hematoxylin and eosin
HSCs	hepatic stellate cells
KEGG	Kyoto Encyclopedia of Genes and Genomes
LF	liver fibrosis
MF	molecular function
NQO1	NAD(P)H quinone oxidoreductase 1
PPI	protein-protein interaction
PSR	Picro Sirius Red
RT-qPCR	Real-time fluorescence quantitative PCR
SASPs	senescence-associated secretory phenotypes
Tregs	regulatory T cells
WB	Western blot
