# Supplementary material for: Bioinformatics-Guided Experimental Validation Identifies NQO1 as a Senescence-Ferroptosis Hub in Liver Fibrosis
Source: Biomedicines. 2025 May 20;13(5):1249. doi: 10.3390/biomedicines13051249 (PMC12108982; doi:10.3390/biomedicines13051249)

Supplementary Figures

Figure S1 The correlation between NQO1 and immune cells. (*P < 0.05, **P < 0.01)

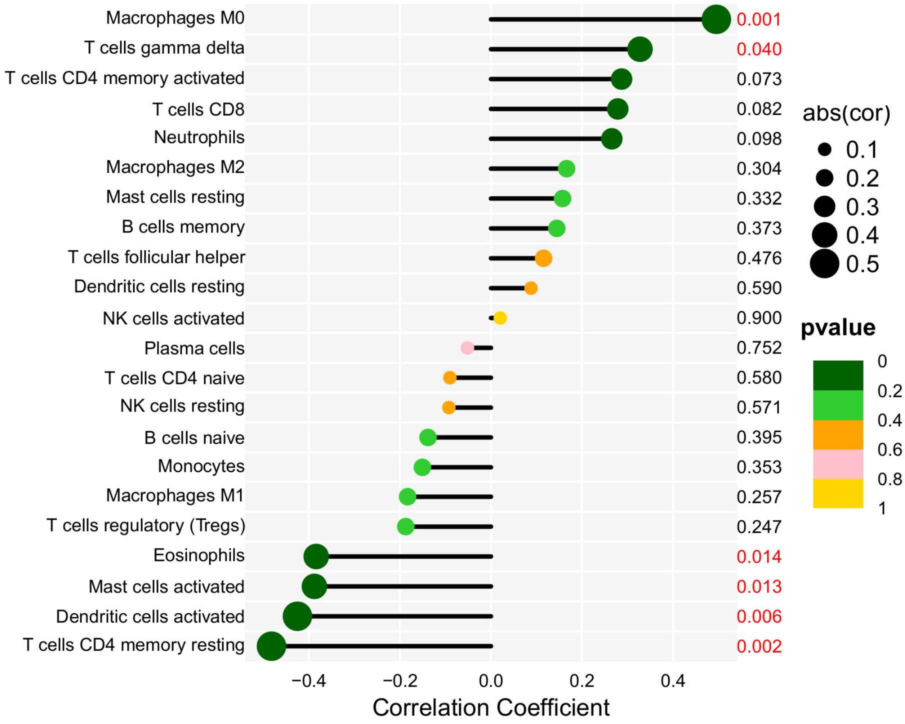

Figure S2 The correlation between AR and immune cells.

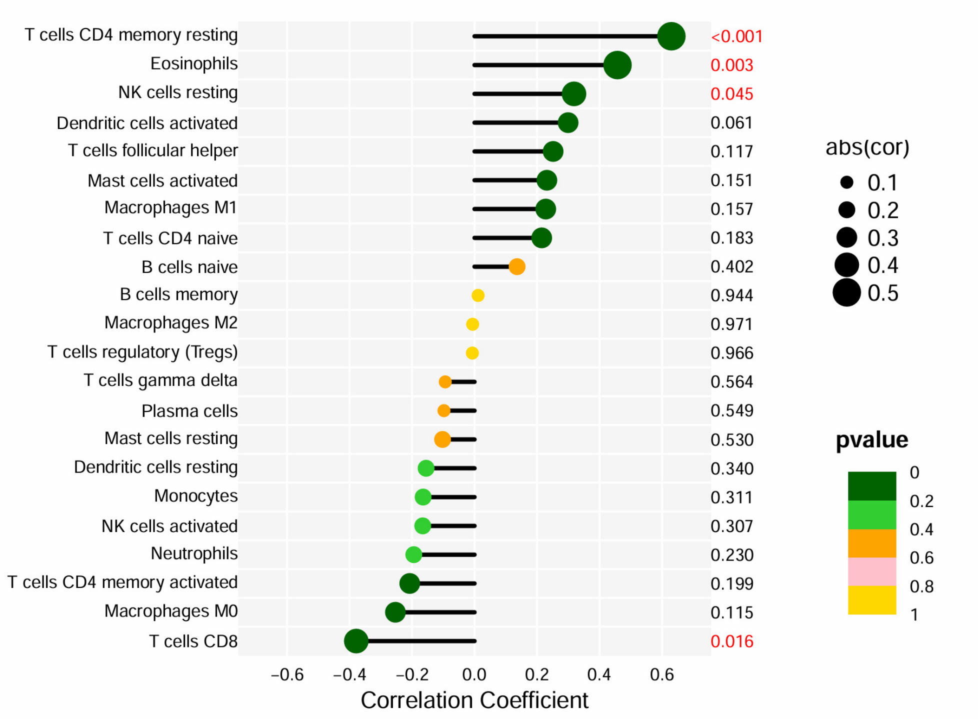

Figure S3 The correlation between CDKN1A and immune cells.

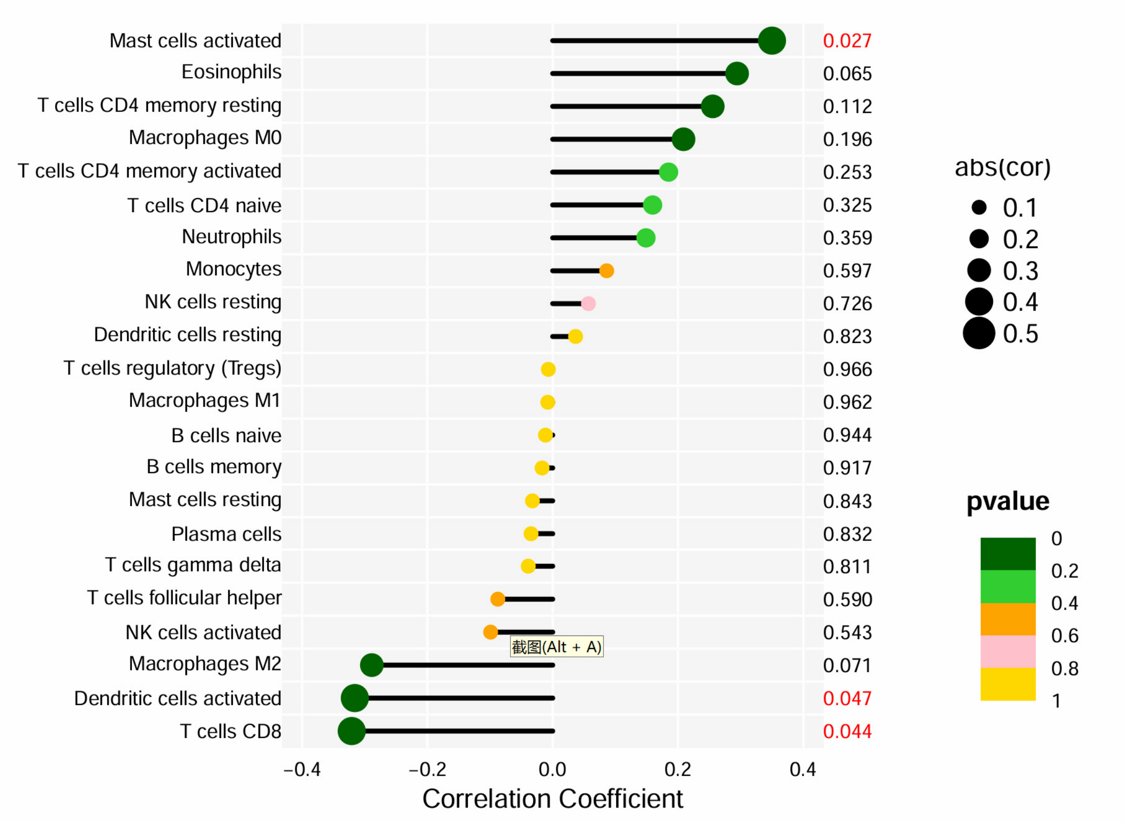

Figure S4 The correlation between GJA1 and immune cells.

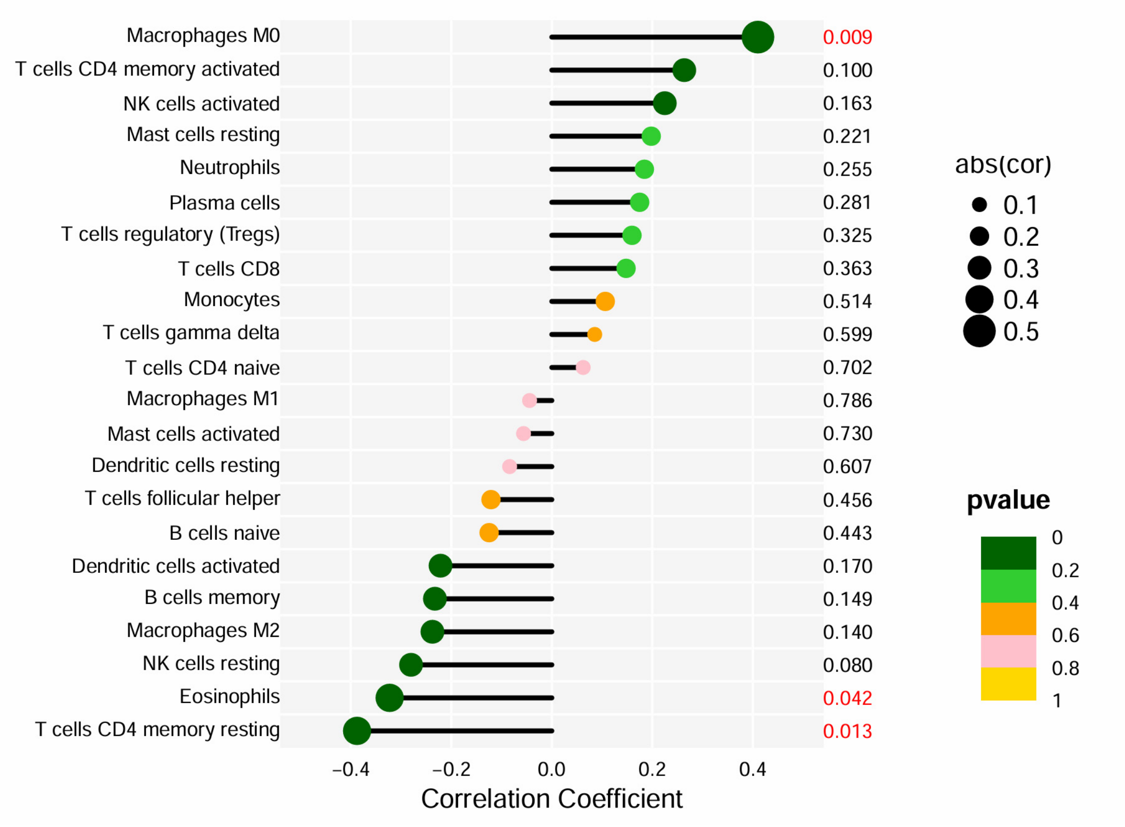

Figure S5 The correlation between CTSB and immune cells.

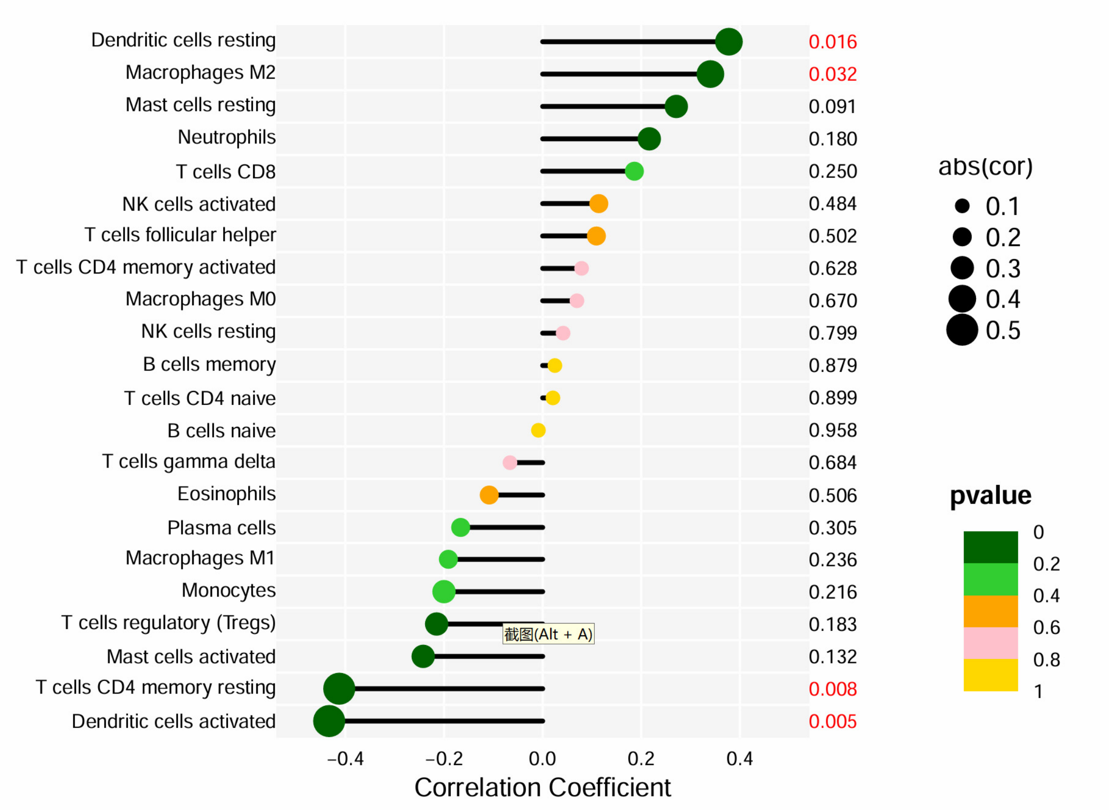

Figure S6 The correlation between HIF1A and immune cells.

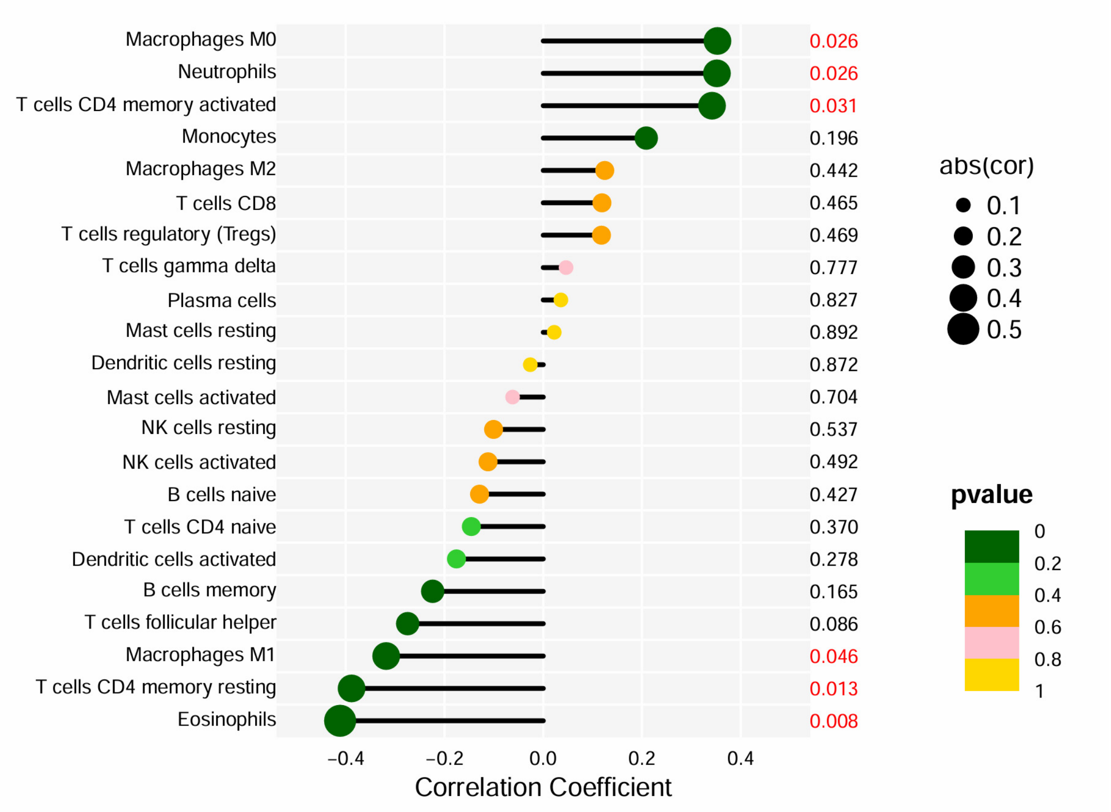

Figure S7 The correlation between HMGB1 and immune cells.

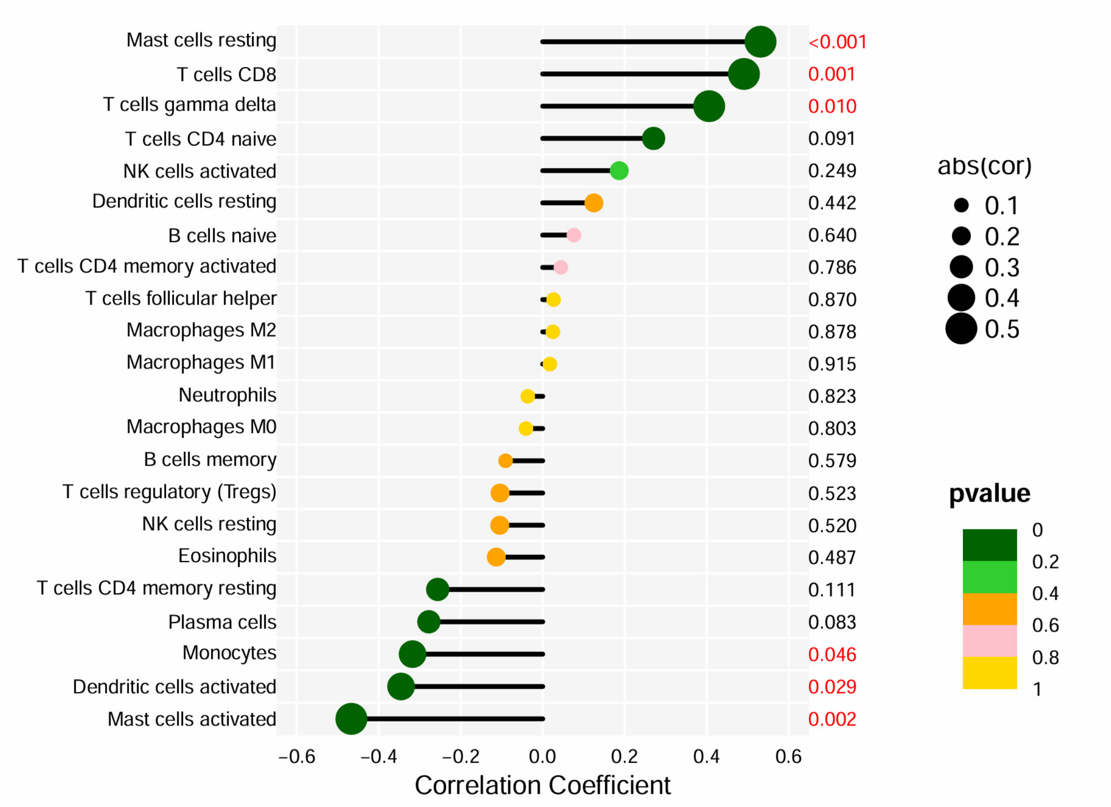

Figure S8 The correlation between PARP1 and immune cells.

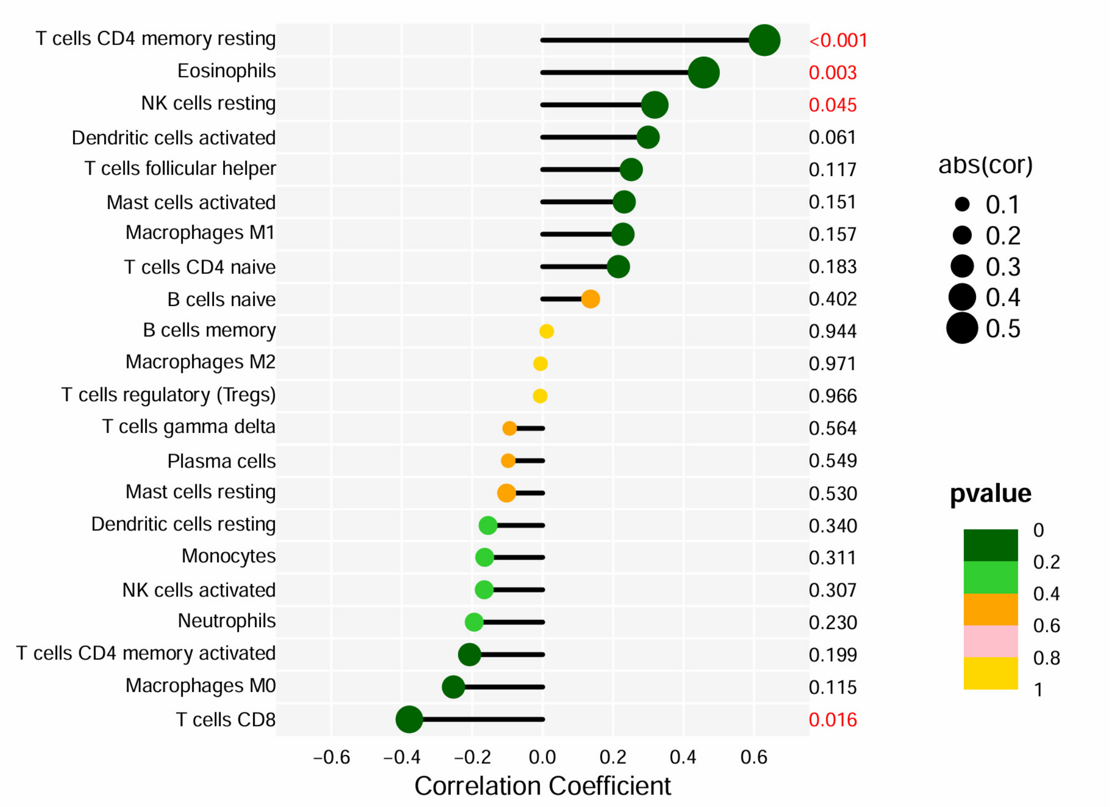

Figure S9 The correlation between PTEN and immune cells.

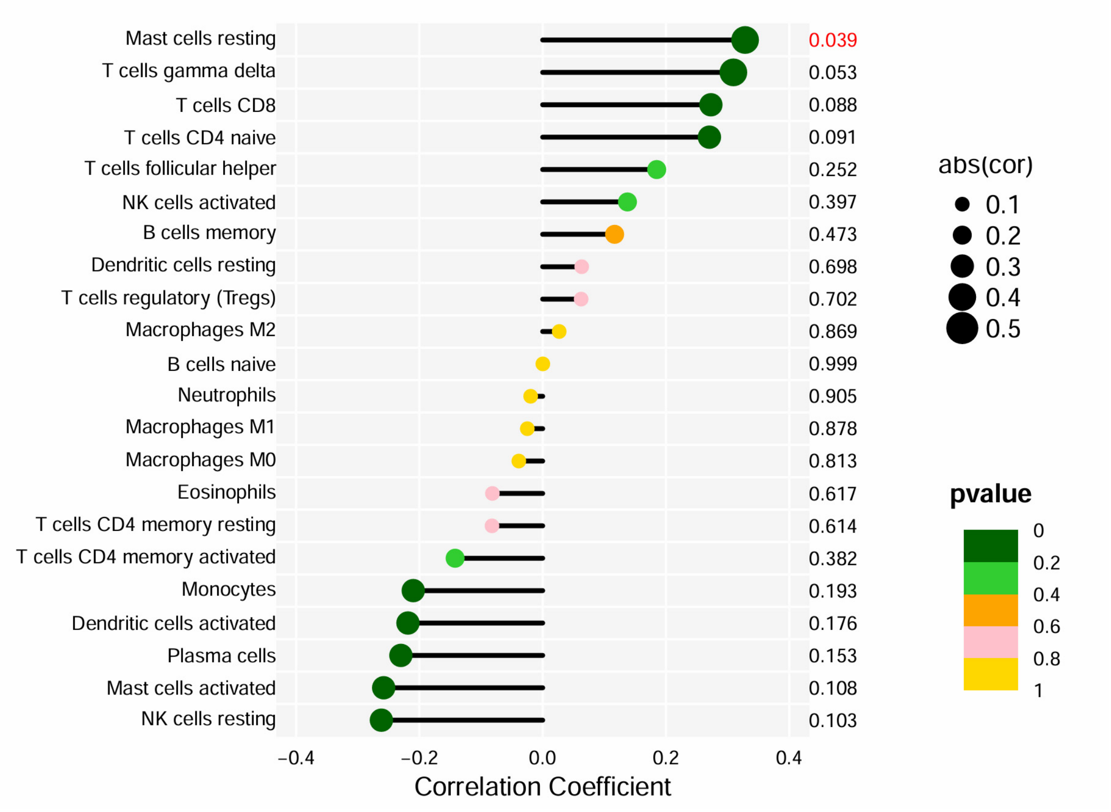

Figure S10 The correlation between TXN and immune cells.

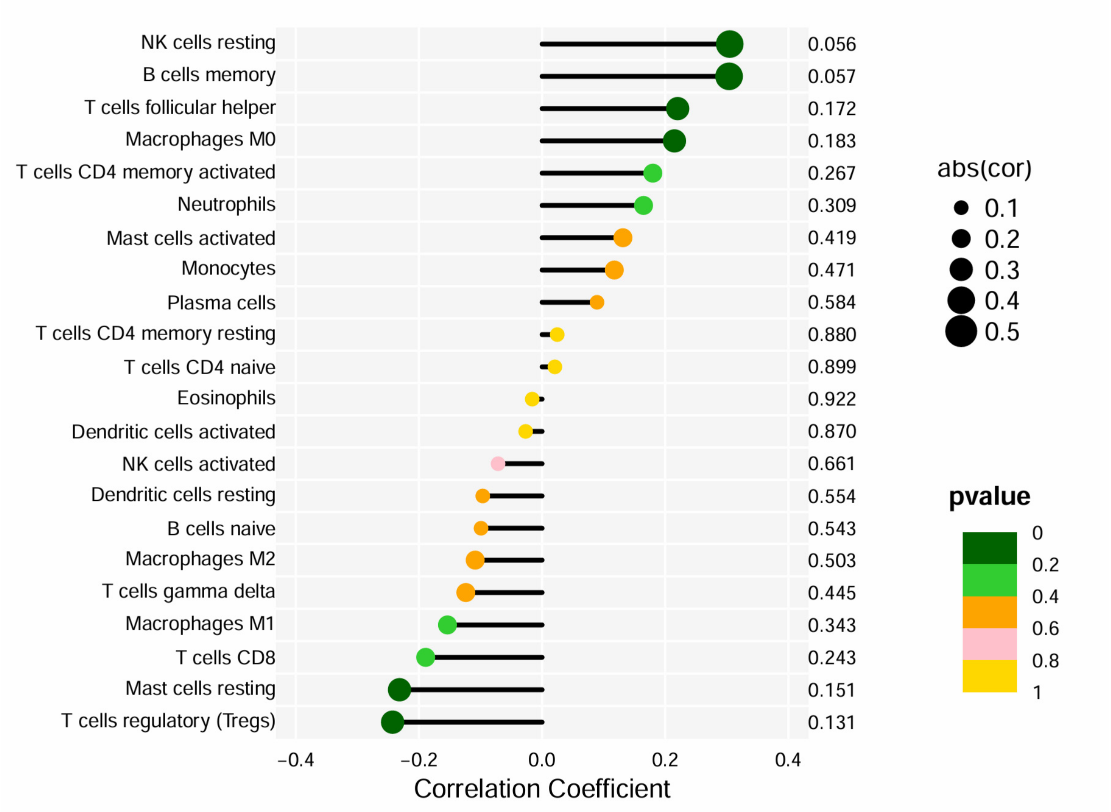

Supplement: Supplementary file 1 [file biomedicines-13-01249-s001.zip › Supplementary Figures.pdf]
