# Supplementary material for: Bioinformatics-Guided Experimental Validation Identifies NQO1 as a Senescence-Ferroptosis Hub in Liver Fibrosis
Source: Biomedicines. 2025 May 20;13(5):1249. doi: 10.3390/biomedicines13051249 (PMC12108982; doi:10.3390/biomedicines13051249)
Supplement: Supplementary file 1 [file biomedicines-13-01249-s001.zip › Supplementary Table S6.pdf]

Supplementary Table S6

The list of siRNA sequence

Genes	Species	Target sequence (5' - 3')
NQO1 siRNA-1	human	CGAGUCUGUUCUGGCUUAUAATT
NQO1 siRNA-2	human	CGAGUGUUCAUAGGAGAGUUUTT
NQO1 siRNA-3	human	UGGAAGAAACGCCUGGAGAAUTT

The list of primers sequence

Genes	Species	Forward primer (5'-3')	Reverse primer (5'-3')
NQO1	human	GCTGGTTTGAGCGAGTGTTC	CTGCCTTCTTACTCCGGAAGG
IL-6	human	CCACCGGGAACGAAAGAGAA	TCACCAGGCAAGTCTCCTCA
CDKN2A	human	GGGGTCGGGTAGAGGAGG	GCCCATCATCATGACCTGGA
TP53	human	AAGTCTAGAGCCACCGTCCA	CAGTCTGGCTGCCAATCCA
α-SMA	human	CGTGCTGGACTCTGGAGATG	GCCCATCAGGCAACTCGTAA
COL1A1	human	CGGTGTGACTCGTGCAGC	ACAGCCGCTTCACCTACAGC
GAPDH	human	CAGCCTCAAGATCATCAGCA	GGTCATGAGTCCTTCCACGA
α-SMA	mouse	CTGACAGAGGCACCACTGAA	CATCTCCAGAGTCCAGCACA
COL1A1	mouse	GAGCGGAGAGTACTGGATCG	GCTTCTTTTCCTTGGGGTTC
NQO1	mouse	TTAAATACACCCTCAGCCCTGG	CGAAGTAACACAATGGGCTTGG
GAPDH	mouse	CTGCGACTTCAACAGCAACT	GAGTTGGGATAGGGCCTCTC

The list of antibody dilution ratio

Antibody	Dilution ratio
NQO1	1:2000
P21	1:1000
P53	1:1000
α-SMA	1:1000
COL1A1	1:1000
Tubulin	1:5000
